# Phospho-Tyrosine(s) vs. Phosphatidylinositol Binding in Shc Mediated Integrin Signaling

**DOI:** 10.4236/ajmb.2015.52003

**Published:** 2015-04

**Authors:** Xiaochen Lin, Olga Vinogradova

**Affiliations:** Department of Pharmaceutical Sciences, School of Pharmacy, University of Connecticut at Storrs, Storrs, USA

**Keywords:** Shc, Integrin, Phosphatidylinositol, ITC, NMR

## Abstract

The Shc adaptor protein, particularly its p52 isoform, has been identified as a primary signaling partner for the tyrosine(s)-phosphorylated cytoplasmic tails of activated *β*_3_ integrins. Inspired by our recent structure of the Shc PTB domain in complex with a bi-phosphorylated peptide derived from *β*_3_ cytoplasmic tail, we have initiated the investigation of Shc interaction with phospholipids of the membrane. We are particularly focused on PtdIns and their effects on Shc mediated integrin signaling *in vitro*. Here we present thermodynamic profiles and molecular details of the interactions between Shc, integrin, and PtdIns, all of which have been studied by ITC and solution NMR methods. A model of p52 Shc interaction with phosphorylated *β*_3_ integrin cytoplasmic tail at the cytosolic face of the plasma membrane is proposed based on these data.

## 1. Introduction

Integrin mediated signaling events control numerous developmental, physiological and pathological processes in multicellular organisms. Although significant progress has been achieved in the understanding of integrin activation [1] [2], the early intracellular events following the integrin mediated extracellular matrix engagement are not well characterized structurally. To illuminate the potential mechanisms of integrin selectivity in the recognition of proximal effectors at different stages of cell spreading, we have studied the effect of phosphorylation on the conformation, membrane insertion, and target binding capability of the cytoplasmic tail of the major platelet integrin *α*_IIb_*β*_3_ [3] [4]. Here we extend this investigation by presenting our new work on deciphering the role that PtdIns lipids might share with phosphotyrosine(s) in integrin signaling.

Amongst the number of potential chemical modifications correlated with the activated state of integrin receptor, it is the tyrosine(s) phosphorylation of the cytoplasmic tail that has been proven crucial for the outside-in signaling events [5]–[9]. Shc p52 isoform, more specifically, its PTB domain, has been identified as the primary signaling partner for the tyrosine(s) phosphorylated *β*_3_CT [9] [10]. This cytoplasmic adaptor protein, known to mediate the MAPK signaling pathway [11], needs to be localized at the cytosolic face of the plasma membrane in order to be readily recruited by a number of different receptors [12]. Two of Shc’s three distinct domains, PTB and SH2, have the potential to bind phospholipids and, thus, might assist Shc translocation to the lipid bilayer [13] [14]. Though only the PTB, not the SH2 domain, is shown to interact with specific PtdIns [15]. The purpose of the work presented here is to compare molecular details of the interaction between Shc and integrin to the binding mode between Shc and phospholipids.

Since their discovery about two decades ago, PTB domains have been identified and structurally characterized within numerous multi-domain proteins. The ways they interact with their targets are strikingly versatile. The phosphorylated tyrosine is not always required for the high affinity interactions, and, in some cases, the binding motif might not even contain the tyrosine residue at all [16]. PTB domains in general are also famous for the significant variance in their ligand binding affinities, ranging from 0.1 to 100 μM [17]. Shc PTB domain in particular exhibits a wide range of affinities to various tyrosine-phosphorylated binding partners. The tightest interaction, with a *K_d_* of 0.19 μM, was measured by ITC for the nerve growth factor receptor TrkA derived peptide [18], correcting the originally over-estimated *K_d_* value of 53 nM (by Scatchard analysis of Surface Plasmon Resonance data) [19]. Upon the structure determination of Shc PTB in complex with a *β*_3_ integrin derived bi-phosphorylated peptide, bi-*β*_3_, [4], we have found that the C-terminal phosphotyrosine (pY759) of *β*_3_CT occupies the classical PTB binding pocket. The other phosphotyrosine (pY^747^) containing motif fits nicely into the grove formed between the second helix and the unusually long flexible loop (~24 residues) connecting the second beta strand with the second helix in PTB fold. In this interaction, a negatively charged phosphate group of pY^747^ forms a salt-bridge with a positively charged side chain of Shc R^104^. This second binding site is in close proximity to the Shc residue R^112^, which is situated at the beginning of the PTB domain’s second beta strand. This R^112^, along with two lysines, K^116^ and K^139^, which also contain positively charged side chains, has been proposed earlier to mediate Shc PTB-phospholipids interaction [20]. In addition, Zhou and co-workers have originally found that Shc PTB domain has higher specificities to acidic PtdIns, particularly PtdIns_(4)_P_1_ and PtdIns_(4,5)_P_2_, over the other lipids that they have tested [15]. Furthermore, the binding of the full length Shc, enclosing the SH2 domain, to Ins_(1,4,5)_P_3_, representing the polar head group of PtdIns_(4,5)_P_2_, has been reported to be much tighter than the binding of the isolated Shc PTB domain alone to PtdIns_(4,5)_P_2_ [21]. Therefore, in order to investigate whether *β*_3_ integrin/phospholipids binding sites on Shc PTB surface indeed overlap, what the thermodynamic profiles of these interactions are, and how *β*_3_CT derived phosphopeptides might affect Shc’s ability to localize itself to the lipid bilayer, we have initiated a through biophysical characterization of the above system. Here we present our data, acquired by ITC and NMR experiments, which allowed us to visualize PtdIns mediated Shc association with the lipid bilayer and how tyrosine(s)-phosphorylated integrin cytoplasmic tail could later replace PtdIns from their binding site on Shc surface. Out data also suggest that the interaction between the cytoplasmic tails of activated integrin receptors and Shc may result in the rearrangement/separation of Shc internal domains.

## 2. Materials and Methods

### 2.1. Materials and Reagents

Ins_(1,4,5)_P_3_ was purchased from Sigma and Cayman Chemical. (dihexanoyl- or dioctonoyl-) PtdIns_(4)_P_1_, PtdIns_(5)_P_1_ and PtdIns_(4,5)_P_2_ were purchased from Cayman Chemical. Phosphopeptides derived from *β*_3_CT(bi-*β*_3_: ^736^RAKWDTANNPL(pY)KEATSTFTNIT(pY)RGT^762^ and c-*β*_3_: 750ATSTFTNIT(pY)RGT762) were synthesized by NEO-Peptide.

### 2.2. Cloning and Protein Purification

The Shc PTB domain (17 – 207) containing the pET15b vector was transformed into a Rosetta (DE3) cell line to pursue optimal expression levels [4]. SH2 domain (380 – 473) was cloned into NdeI and XhoI sites in pET21b vector and then transformed into a BL21 (DE3) cell line. The vector of pET28a-full length Shc (1 – 473), generously provided by Dr. Ladbury (University of Texas MD Anderson Cancer Center, TX), was expressed in a BL21 (DE3) cell line as well. Typically, 10 ml overnight culture was used to inoculate 1 liter LB broth. Cells were grown at 37°C until OD_600_ reached 0.6. At this point the culture was cooled to room temperature and then the overnight overexpression was induced by 1 mM isopropyl-*β*-D-thiogalactopyranoside. Harvested cells were kept at −20°C for future purification or resuspended in lysis buffer supplemented with protease inhibitors. Cells were further lysed by french press and the cell debris was removed by centrifugation at 18,000 rpm at 4°C. The N-terminal His-tag fused PTB domain and full length Shc, and the C-terminal His-tag fused SH2 domain were first purified under native conditions with Ni-NTA resin (QIAGEN) using the standard protocol followed by a buffer exchange to 50 mM Tris, pH 7.5, 100 mM NaCl, 5 mM *β*-mercaptoethanol in a Superdex-75 column (GE Healthcare) where all three recombinant proteins showed a proper folding in monomeric state (Supplementary Figure S1). The overexpression of the target protein and the purified protein were confirmed by SDS-PAGE and the bands of the expected mass were observed. The determination of protein concentration by UV measurement was consistent with Bradford assay.

### 2.3. ITC Experiments

Calorimetric measurements of a full length Shc, PTB domain, and SH2 domain to *β*_3_ phosphopeptides or PtdIns were performed on a low volume Nano ITC (TA Instruments). All experiments were carried out in a buffer of 50 mM Tris, pH 7.5, 100 mM NaCl, 5 mM *β*-mercaptoethanol (unless described otherwise) at 12°C or 25°C with a stirring speed of 250 rpm to minimize the precipitation of the protein. 300 second time intervals were set between injections. The concentration of protein samples was determined by UV measurements. The titrants (*β*_3_ phosphopeptides or PtdIns) were prepared in the same buffer by the dilution of higher concentration stocks. The concentrations of the protein ranged from 30 μM to 150 μM. The concentration of protein was examined after each ITC experiment by UV to ensure the stability of proteins under the test condition. The analysis of the data was done with Nano Analyze Software (TA Instruments) suite using an “independent” model. In all cases, a stoichiometry of 1 ± 0.15 was revealed for the interaction between protein and *β*_3_ phosphopeptides/PtdIns.

### 2.4. ^15^N-HSQC Titration by NMR

^15^N-labelled recombinant proteins were overexpressed in M9 medium supplemented with ^15^NH_4_Cl as the sole nitrogen source. The same purification procedures as described above were followed. NMR samples of 100 – 200 μM were prepared in a buffer of 50 mM Tris, pH 7.5, 100 mM NaCl, 5 mM *β*-mercaptoethanol, 7% D_2_O, 1 mM 4,4-dimethyl-4-silapentane-1-sulfonic acid as the internal standard. ^15^N-HSQC titration experiments were performed on a Varian/Agilent Inova 600 MHz spectrometer equipped with inverse-triple resonance cryo-probe at 35°C. All spectra were processed with NMRPipe [22] and analyzed in CCPNmr Analysis software suite [23].

## 3. Results

### 3.1. Shc Interaction with Phosphatidylinositols Tested Using Ins_(1,4,5)_P_3_

We started our investigation by studying Shc binding to Ins_(1,4,5)_P_3_ using ITC. We were greatly surprised that we were not able to reproduce the data reported by George and co-workers, who have reported a tight dissociation constant (*K_d_* of 0.7 μM) for the full length Shc [21]. No binding was found under any conditions tested (to have a better control on all possible variables, we have purchased Ins_(1,4,5)_P_3_ from the same supplier (Cayman Chemical) as reported in the paper). The conditions we tested include: i) Shc PTB domain at 12°C and 25°C in several different buffers (including exactly the same as the one described by George, which is 50 mM Tris, pH 8.0, 200 mM NaCl, 1 mM Dithiothreitol); ii) Shc full length at 12°C and 25°C; and iii) Shc SH2 domain at 12°C and 25°C (typical raw titration data is shown as an example in the Supplementary Figures S2(a)–(c)). In the attempt to interpret our negative findings, we hypothesized that the heat of the reaction was too small, actually below the detection range of our NanoITC calorimeter. Thus, we turned to NMR as the method of choice due to its endogenous ability to capture the interactions with minimal or no enthalpy changes. In this case, we performed ^15^N-HSQC chemical shifts mapping experiments by titrating the unlabeled Ins_(1,4,5)_P_3_ into the ^15^N-labled Shc PTB domain. Under the conditions tested (see Materials and Methods for details), we only observed minimal chemical shift perturbations for a handful of residues in the ^15^N-HSQC spectra even at the maximum of lipid to protein ratio (15 to 1, Supplementary Figure S2(d)). Uncertain about these findings, we decided to choose different lipid mimetics for further investigation.

### 3.2. Shc Interaction with Phosphatidylinositols Tested by ITC Using PtdIns_(4)_P_1_ and PtdIns_(4,5)_P_2_

With minimal success observing Shc interaction with Ins_(1,4,5)_P_3_, representing exclusively the polar head-groups of phosphatidylinositols, we wondered whether the aliphatic chains of the lipids were necessary for the measurability of the binding by ITC and NMR. Long lipid chains are not exactly soluble in aqueous solution. Thus, to perform the binding studies described below, we restricted the length of aliphatic chains to six carbons in dihexanoyl, or eight in dioctonoyl phosphatidylinositol derivatives and concentrated on PTB domain of Shc, previously shown to interact specifically with PtdIns_(4)_P_1_ and PtdIns_(4,5)_P_2_ [15]. Binding affinities for the apo PTB domain, originally examined in the unilamellar liposomes centrifugation assay, were reported to be 52 μM for PtdIns_(4)_P_1_ and 140 μM for PtdIns_(4,5)_P_2_. Binding affinity of the PTB domain complexed with TrkA peptide was reported to be even weaker, with a roughly estimated *K_d_* of 450 μM for both lipids.

**Figure 1:**
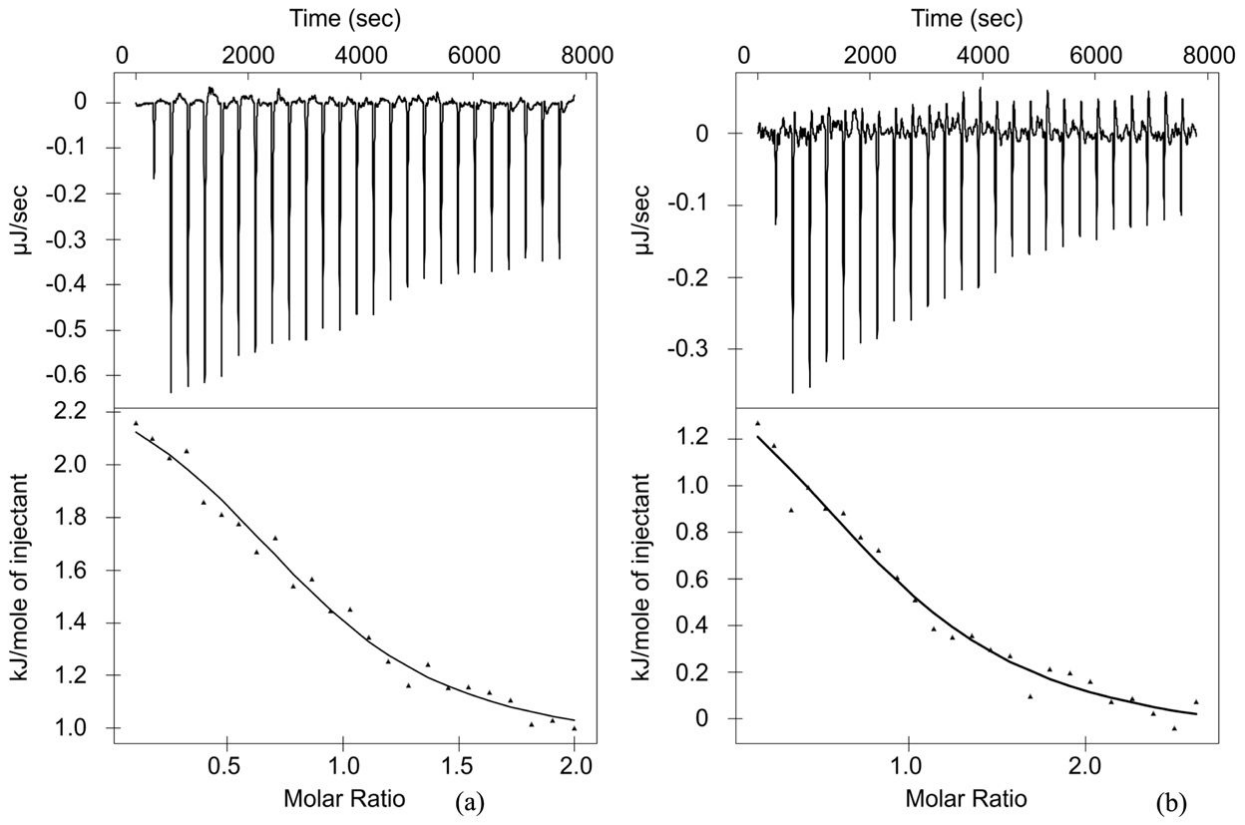
(a) PtdIns_(4)_P_1_ and (b) PtdIns_(4,5)_P_2_ binding to Shc PTB domain at 25°C.

First, we employed ITC to determine the binding affinities of the PTB domain to three different phosphatidylinositol containing soluble probes (see the Materials and Methods section for details). We measured a *K_d_* of 95 μM and 125 μM for PtdIns_(4)_P_1_ and PtdIns_(4,5)_P_2_ respectively (titration curves and isotherms are presented in Figure 1(a) and Figure 1(b)). ITC was not able to detect the binding PtdIns_(5)_P_1_ (titration curve is shown in the Supplementary Figure S3), thus confirming the specificity of interaction reported by Zhou and coworkers. The ITC measurements demonstrated similar binding affinity or PtdIns_(4,5)_P_2_ as compared to the affinity previously determined by centrifugation assay data analysis, though PtdIns_(4)_P_1_ appeared to bind about two times weaker than reported before [15]. Small amounts of heat are consumed upon binding (in addition to the relatively large heat of solvation), demonstrating endothermic interactions at 25°C that are driven predominantly by entropy. Considering these data, it is not entirely surprising that we were only able to observe minimal Shc PTB binding with Ins_(1,4,5)_P_3_ alone in ITC. The complete thermodynamic analysis of the above interactions is presented in the Table 1.

### 3.3. PtdIns Binding Sites on Apo Shc PTB Surface Partially Overlap with Integrin Binding Sites

**Figure 2:**
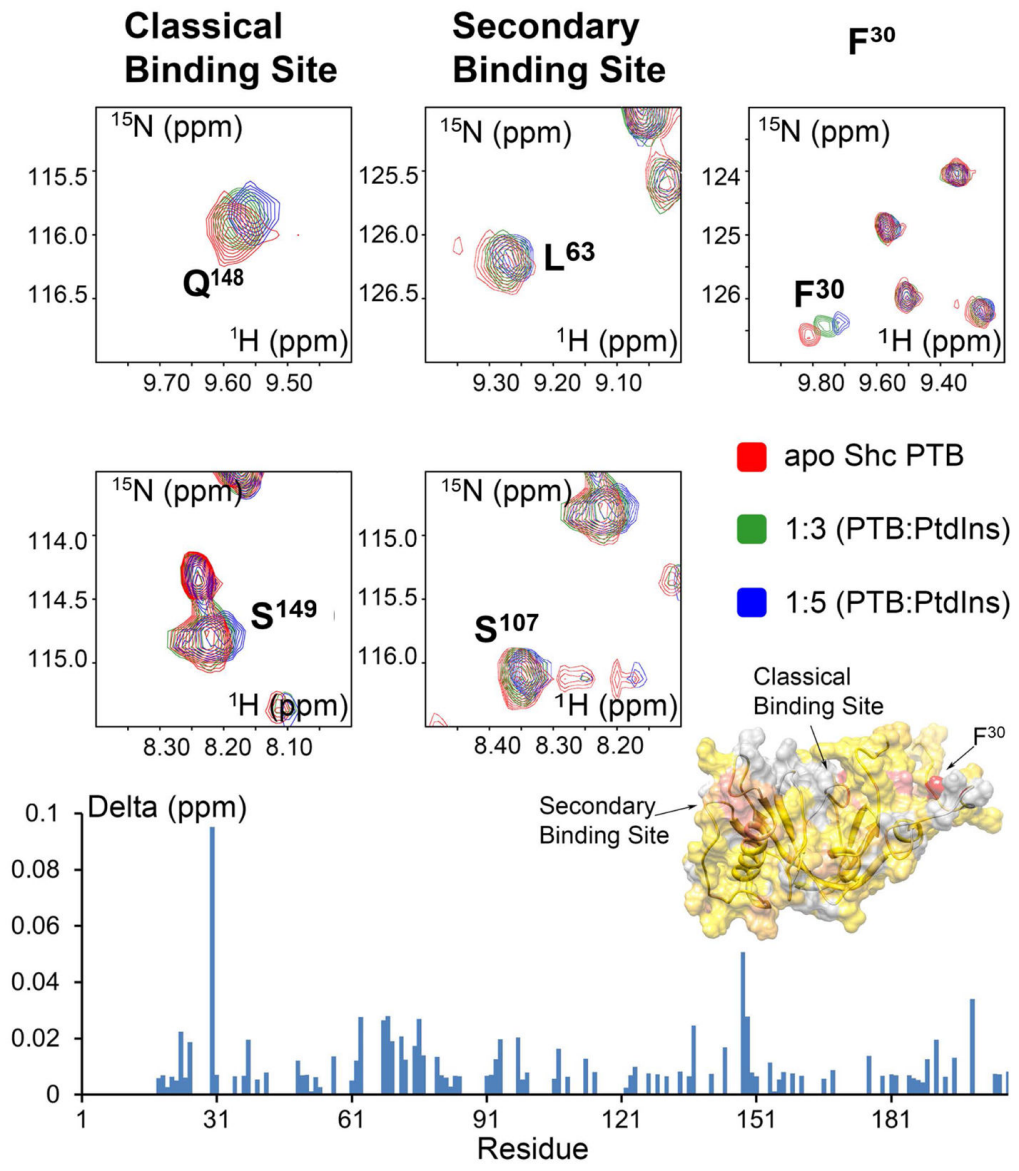
^15^N-HSQC spectra showing chemical shifts perturbations in a classical PTB binding pocket, the second binding site and F^30^. Chemical shifts perturbations upon PtdIns_(4,5)_P_2_ binding to apo Shc PTB domain inset with surface mapping of PTB generated from PDB entry 1OY2. The surface is colored according to the corresponding chemical shift perturbations, delta (ppm), from yellow (smallest) to red (largest) and light gray indicating missing assignments. Delta (ppm) refers to the combined HN and N chemical shift changes, obtained from the equation: Δ*δ*(HN, N) = ((Δ*δ*HN^2^ + 0.2(Δ*δ*N)^2^)^1/2^, where Δ*δ* = *δ*_bound_ – *δ*_free_.

To find out the molecular details of these weak Shc-PtdIns interactions, we next employed NMR and performed ^15^N-HSQC chemical shifts mapping experiments which are especially useful to define weak binding [24]. Two non-labeled PtdIns derivatives, PtdIns_(4)_P_1_ and PtdIns_(4,5)_P_2_, demonstrated to interact with Shc PTB by ITC experiments, were titrated into the samples containing ^15^N-labeled apo PTB domain. Chemical shift perturbations, associated with the probes binding, were monitored. Overall, the observed shifts in Shc PTB resonance frequencies were small. However, they were concentration dependent, reproducible, and similar among the two PtdIns tested. Furthermore, the same residues were affected as the ones in Ins_(1,4,5)_P_3_ titration described above (Supplementary Figure S2(d)), but the shifts were more pronounced in case of PtdIns_(4,5)_P_2_, even at 5 to 1 lipid to protein ratio as compared of 15 to 1 ratio for Ins_(1,4,5)_P_3_. The close-ups of 15N-HSQC spectra of PTB in the presence of PtdIns_(4,5)_P_2_, exemplifying the most shifted resonances, are shown in Figure 2. These chemical shifts perturbations occur at three major binding regions. The first one, represented by the residues Q^148^, S^149^ and A^168^, overlaps with the classical phosphotyrosine binding pocket of the protein-peptide binary complex. However, the canonical PTB fold cannot be formed without the stabilizing hydrophobic residue (pY-5) of the target peptide. Thus, in apo PTB domain Ins_(1,4,5)_P_3_ could only establish intermediate dynamic contacts with the amine functional groups of R^67^ or Q^148^ side chains. The second binding site, including the residues L^63^, Q^76^ and S^107^, is formed at the interface of the second helix, the loop connecting the first beta strand with the second helix and the beginning of the second beta strand. This second PtdIns binding site is distinct from the second novel phosphotyrosine binding site we previously described for Shc PTB-bi-*β*_3_ complex [4], although it includes several overlapping residues. The third PtdIns binding site is restricted to a single but the most perturbed residue of the spectra, F^30^, which is located within the unstructured Shc PTB N-terminus. The chemical shift perturbations in ^15^N-HSQC spectrum of apo Shc PTB upon PtdIns_(4,5)_P_2_ titration, plotted against the residue number, are presented in Figure 2. The inset demonstrates affected residues mapped onto PTB surface, which is colored according to the absolute value of the corresponding chemical shift perturbations.

### 3.4. Integrin Interactions with Shc-PTB Domain vs. Full Length Shc Story

**Figure 3:**
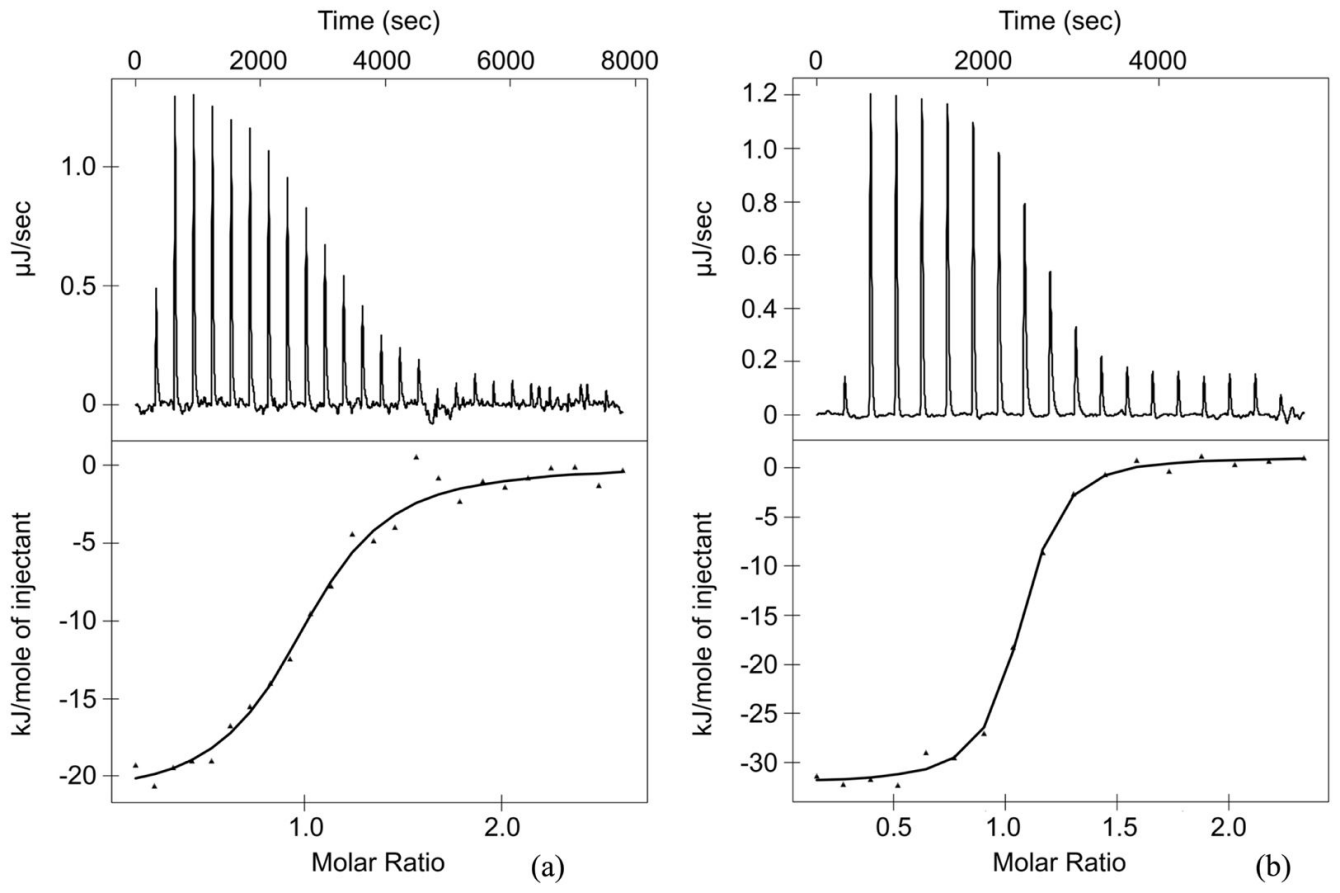
(a) Bi-*β*_3_ peptide and (b) c-*β*_3_ peptide binding to Shc PTB domain at 25°C.

Two of the Shc three distinct domains, PTB and SH2, have the potential to bind phosphotyrosines and/or phospholipids. However, in all the experiments performed we did not observe any indications of Shc SH2 domain interaction with either PtdIns or *β*_3_ integrin derived tyrosine(s)-phosphorylated peptides. Thus, we focused our investigation on studying the PTB domain alone and comparing it to the full length Shc in order to understand the thermodynamic forces driving the interactions. We started with a bi-*β*_3_ peptide, which was used previously for the structural characterization of the Shc PTB/integrin binary complex [4]. We found that at 25°C the *K_d_* of this interaction was 5.1 μM (Δ*G* = −30.17 kJ/mol) and it was predominantly driven by enthalpy (Δ*H* = −22.98 kJ/mol). A representative calorimetric isotherm and the corresponding titration curve, depicted in Figure 3(a), demonstrate that the interaction is exothermic at 25°C, releasing the heat upon peptide binding with a stoichiometry about 1. The driving force of this complex formation is very different from the one defined for the TrkA peptide (enthalpy vs. entropy), and it shows a higher specificity, even though the binding affinity is about twenty five times weaker (5.14 μM vs. 0.19 μM). We have speculated that the secondary binding site for pY^747^ within the complex might be responsible, at least in parts, for the difference. To find out whether this was the case, we ran ITC experiments for the smaller c-*β*_3_ peptide containing only one terminal pY^759^ (Figure 3(b)). Our reasoning appeared to be wrong. The shorter (13 vs. 27 residues) mono-phosphorylated peptide had an even higher binding affinity with the *K_d_* of 0.34 μM (Δ*G* = −36.86 kJ/mol) and an even larger favorable enthalpy term (Δ*H* = −25.41 kJ/mol). The thermodynamic analysis of the above interactions is presented in Table 2.

Next, we decided to find out how the Shc solvent-accessible surface area was affected during the reaction. This was accomplished by investigating the change in constant pressure heat capacity (Δ*Cp*). Δ*Cp* is given by the slope of the linear regression analysis of Δ*H* plotted vs. temperature, and it is often independent of temperature within the narrow physiological range (reviewed in [25]). Thus, two temperature points, 12°C and 25°C, would provide an estimate for the slope. For binding reactions, negative Δ*Cp* is expected to demonstrate the reduction in protein surface area in contact with the solvent. The larger and more negative values reflect a larger surface area buried upon complex formation. Through this correlation, structural information of the macromolecular complex could be inferred from the thermodynamic parameters. As expected, the longer bi-*β*_3_ peptide in complex with Shc PTB domain demonstrated a more negative Δ*Cp* of −0.88 kJ/mol·K as compared to the shorter c-*β*_3_ peptide represented by Δ*Cp* of −0.09 kJ/mol·K (see Table 2 for details). This correlates well with the larger solvent-accessible surface area buried upon binding of the longer peptide. As we already established that the Shc SH2 domain does not bind to either of the above peptides, we decided to confirm that the full length Shc behaves similarly to PTB domain alone. To our great surprise that was not the case. We found that while bi-*β*_3_ peptide in complex with full length Shc showed a quite similar Δ*Cp* of −0.96 kJ/mol·K, binding of c-*β*_3_ peptide resulted in a *positive* Δ*Cp* of 1.04 kJ/mol·K. That means that upon complex formation with c-*β*_3_ peptide more of the Shc solvent-accessible surface area was exposed. The only reasonable explanation of this phenomenon that we can imagine is the unraveling of the intramolecular interactions between the domains within Shc itself.

### 3.5. PtdIns Only Weakly Compete with *β*_3_-Peptides for Interaction with Shc

**Figure 4:**
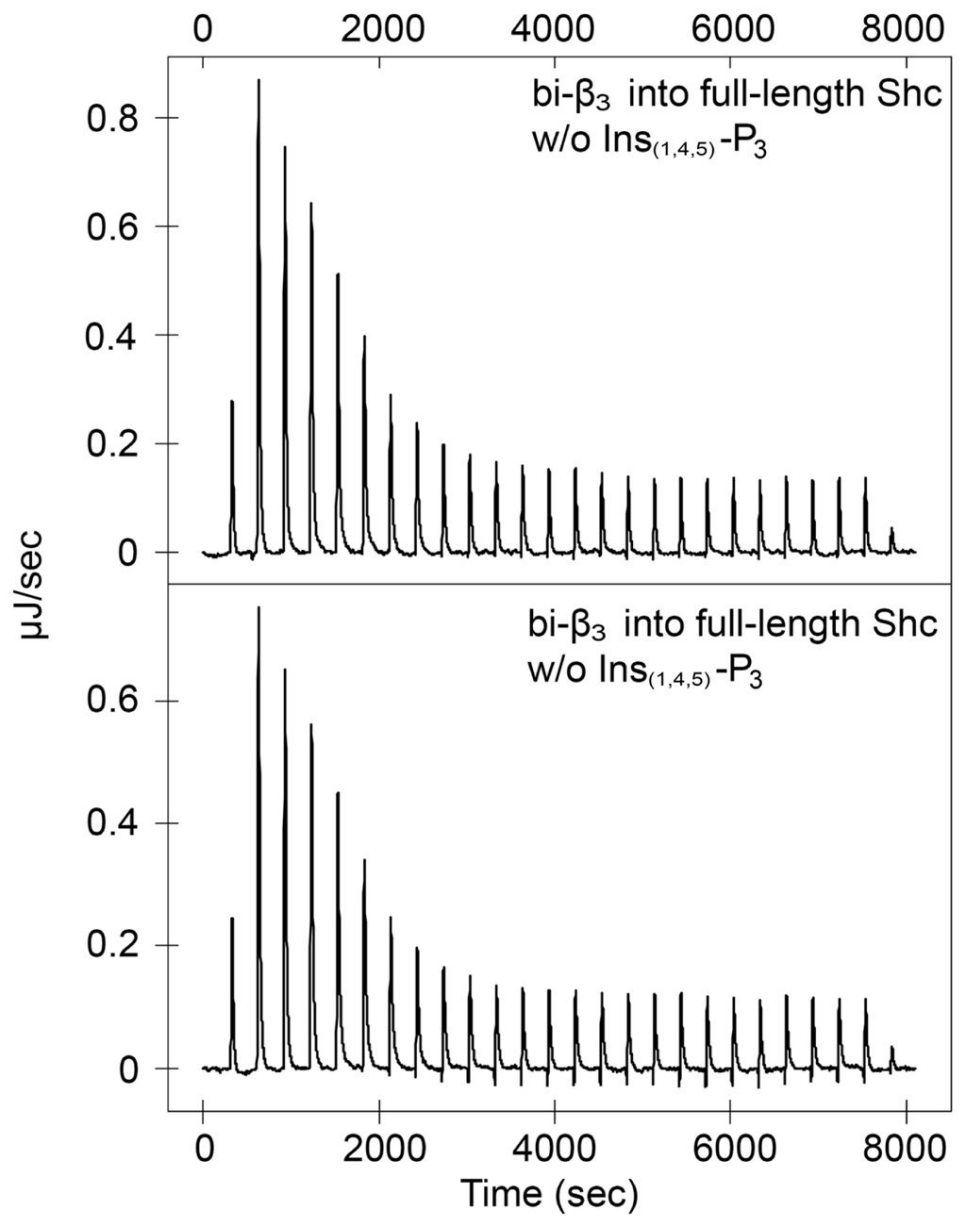
Full length Shc binding to bi-*β*_3_ in the absence (upper) and presence (lower) of Ins_(1,4,5)_P_3_ at 25°C.

We were also curious about the relation between binding sites for PtdIns and *β*_3_-peptides (bi-*β*_3_ and c-*β*_3_) on Shc PTB surface. If these sites do not overlap, the presence of *β*_3_-peptide should not interfere with the interaction between PTB and PtdIns; thus, PtdIns should cause concentration-dependent perturbations in the spectra of the PTB-*β*_3_-peptides complex. In case of a competitive binding between PtdIns and *β*_3_-peptides to PTB, since Shc’s affinity to bi-*β*_3_ and c-*β*_3_ is much higher in comparison to its affinity to phospholipids, it is reasonable to speculate that integrin tyrosine(s)-phosphorylated CTs can easily replace PtdIns from the partially overlapping binding sites on the Shc surface. We have proved the latter scenario by using NMR. First, we prepared protein samples of the PTB domain with PtdIns added at protein-PtdIns ratios of 1:0, 1:3, 1:5 and 1:15. Then bi-*β*_3_ or c-*β*_3_ was added to the sample at a 1:2 protein-to-peptide ratio and ^15^N-HSQC experiments were performed. The ^15^N-HSQC spectra of both Shc PTB-bi-*β*_3_ and Shc PTB-c-*β*_3_ complexes in the absence and the presence of PtdIns_(4)_P_1_, PtdIns_(4, 5)_P_2_ or Ins_(1, 4, 5)_P_3_ overlap perfectly (an example of the overlapping spectra is presented Supplementary Figure S4). Therefore, we have confirmed that the binary interfaces between Shc PTB and phosphopeptides remained the same in the presence of phospholipids added in excess. Also, bound peptides did not allow PtdIns to occupy their partially accessible (according to the chemical shifts mapping experiments summarized in Figure 2) potential binding sites on the surface of PTB domain. The remaining question was whether PtdIns could affect the thermodynamic profiles of Shc interaction with phosphorylated peptides. To test this, we investigated the binding of full length Shc and Shc PTB domain to bi-*β*_3_ and c-*β*_3_ peptides in the presence of Ins_(1,4,5)_P_3_ by ITC. As expected, our data indicate that the protein samples over-saturated by Ins_(1,4,5)_P_3_ at 3 to 1 lipid to protein ratio were not much different in Shc binding to either of the peptides. The most noticeable difference, observed in a single case, was the perturbation in the thermodynamic profile of the full-length Shc titrated by bi-*β*_3_, where the unfavorable 3 kJ/mol change in enthalpy was almost compensated by the favorable change in entropy term, resulting in a slight reduction of *K_d_* value (see Table 3). The impact from Ins_(1,4,5)_P_3_ on the peptide interaction with Shc was also evidenced in this case by the appearance of small negative peaks ahead the dominating positive peaks in the titration curve (presented in Figure 4), when the reaction was close to saturation. In the absence of the lipid mimetic, the small negative peaks were not present. This phenomenon is quite specific, as no similar patterns were witnessed in any other ITC experiments performed for studying Shc-bi-*β*_3_/c-*β*_3_ binding. Lastly, to complete the investigation and to confirm the inability of PtdIns to replace the phosphopeptides, we performed titrations of PtdIns into Shc PTB complexed with phosphorylated peptides (bi-*β*_3_ or c-*β*_3_, at a 2:1 peptide to protein ratio). We then monitored potential changes in HSQC spectra by NMR or in calorimetric isotherms by ITC. As expected, no indications of binding were observed by either method.

## 4. Discussion

Scaffold adaptor protein Shc can be recruited through numerous receptors, including integrins, growth factors, antigens, cytokines, G-protein-coupled, and hormone receptors [12]. Interactions of this cytoplasmic protein with specific phospholipids have been proposed as a mechanism for its translocation to the membrane [13] [14]. Despite vast structural, functional, and biophysical data characterizing Shc involvement in signal transduction from different receptors, its interactions with integrins or specific phospholipids are much less studied or understood. We have analyzed Shc binding to tyrosine(s) phosphorylated peptides derived from *β*_3_ integrin and PtdIns using ITC and solution NMR methods. Through these studies we have found that PtdIns only weakly compete with the phosphorylated integrin cytoplasmic tail for Shc PTB binding. This conclusion is based on several observations. First, Shc interactions with phosphopeptides are characterized by several orders of magnitude higher affinity than its binding to PtdIns. Second, Shc interaction with peptides is enthalpy driven in contrast to the entropy driven interaction with phospholipids. Third, the overall conformational rearrangement of Shc PTB domain upon interaction with the phosphopeptides is coupled with the replacement of PtdIns from their binding sites on Shc surface. Lastly, PtdIns cannot replace phosphotyrosines from their PTB binding sites and the unoccupied surface is not sufficient to stabilize Shc PTB interaction with phospholipids.

**Figure 5:**
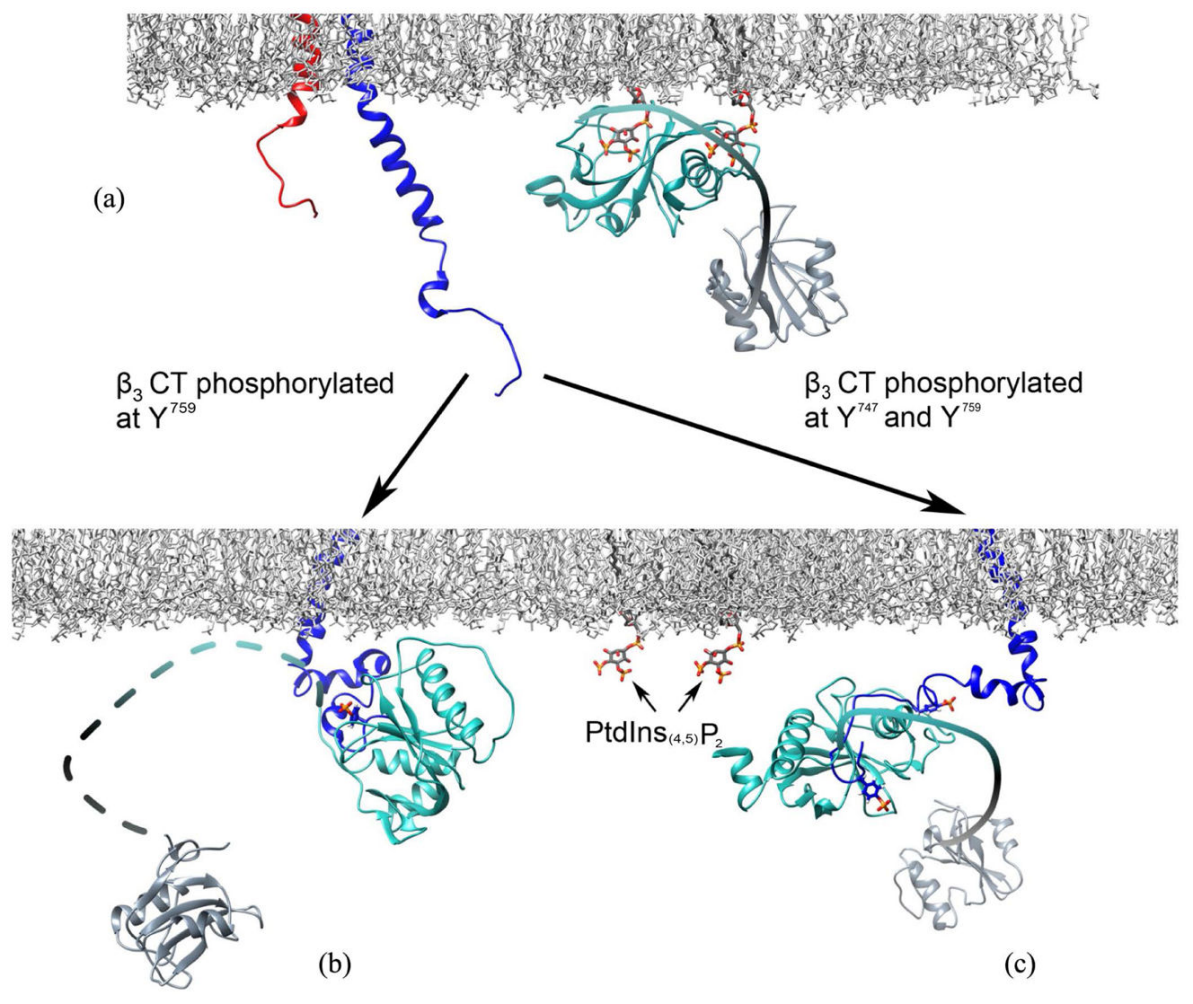
A model for Shc-mediated integrin signaling at the cytosolic face of the plasma membrane. The cytoplasmic tails of integrin *α*_IIb_ and *β*_3_ are shown in red and blue, respectively. The PTB domain and SH2 domain of Shc are shown in cyan and gray, respectively. The molecules of PtdIns_(4,5)_P_2_ and phosphotyrosines are colored according to heteroatoms. This descriptive model was generated in Chimera [28] by using PDB entries: 1OY2, 1TCE, 1S4X, 2KNC, 1SHC, 2LJE, 2L1C, and a phosphorylethanolamine/phosphocholinelipid membrane [29]. (a) Shc is recruited to the membrane by the interaction of its PTB domain with PtdIns, and the integrin heterodimer is shown in a latent state; (b) Binding to the activated mono-phosphorylated *β*_3_CT via PTB results in the conformational rearrangements leading to a more “extended” full length Shc; (c) Binding to the activated bi-phosphorylated *β*_3_CT releases the PTB domain from the membrane, though the *β*_3_CT-PTB complex may still provide the surface area necessary for the interaction with the other internal Shc domains.

The most interesting finding in this study comes from the thermodynamic analysis of mono- and bi-phosphorylated peptides binding to Shc PTB domain vs. the full length Shc. Although there are not many differences in the Δ*Cp* of bi-*β*_3_ binding, characterized by expected negative values about 0.9 kJ/mol·K for solvent-accessible surface area buried in both cases, there is a striking difference in Δ*Cp* of c-*β*_3_ binding. While for the PTB domain alone Δ*Cp* is expectedly reduced (shorter peptide) but still negative, full length Shc binding is characterized by a positive Δ*Cp* value about 1.0 kJ/mol·K. This suggests exposure of the originally buried solvent-accessible surface area, which is larger than the surface of interaction with mono-phosphorylated peptide, and this is only possible if the interactions among PTB, CH1 and SH2 domains within Shc have been disrupted. One of the potential rearrangement schemes for Shc domains upon binding different peptides is proposed in Figure 5. In this scenario, Shc is recruited to the membrane by the interaction of its PTB domain with PtdIns. While binding of the bi-phosphorylated integrin releases PTB domain from the membrane, *β*_3_CT-PTB complex may still provide the surface area necessary for intra-molecular interaction with other domains of Shc. Contrarily, binding of mono-phosphorylated peptide might result in conformational rearrangements leading to a more “extended” Shc structure as PTB domain detaches from the lipid bilayer. As Shc is known to be a positive regulator in the MAPK pathway, the recruitment of Shc to the activated receptors leads to phosphorylation in the CH1 region at Y^239/240^ and Y^317^. The phosphorylated tyrosine residues form two consensus binding motifs to the downstream adaptor protein Grb2 and the formation of Shc-Grb2 complex eventually leads to the activation of the MAPK pathway. We have established previously that integrin cytoplasmic tails are capable of accommodating different structural features depending upon their binding partner and/or the phosphorylation state [1] [3] [4] [26]. Our new data suggests that they can also cause variable conformational rearrangements in their targets, as Shc seems to adapt different conformations when binds mono/bi-phosphorylated *β*_3_ cytoplasmic tail. The plausible biological significance of the dexterity in the conformation of Shc may contribute to differential phosphorylation in the CH1 region. Although both Y^239/240^ and Y^317^ are capable of serving as the binding site for Grb2, the biological outcomes emanating from the two phosphorylation sites can be significantly different [27]. Deciphering this remarkable dexterity should definitely aid in better understanding of crucial bidirectional information flow through these distinct receptors.

## 5. Conclusion

We have: i) confirmed that Shc interacts with both, integrin tyrosine(s)-phosphorylated cytoplasmic tails and PtdIns lipids, through the same PTB domain (another potential hub, SH2 domain, is not involved in these interactions); ii) found that phosphorylated peptides binding is enthalpy driven and tighter, while Shc interactions with PtdIns are entropy driven and are much weaker; iii) determined that Shc interactions with PtdIns and *β*_3_-derived peptides are only weakly competitive and are characterized by partially overlapping bindings sites; iv) observed thermodynamic indications for potential intramolecular interactions within Shc, which could be perturbed by the binding to the phosphorylated receptor; and v) proposed a model for Shc-mediated integrin signaling through its recruitment to the lipid bilayer, which paves the foundation for the future experiments.

## Supplementary Material



## Figures and Tables

**Table 1:** ITC data of Shc PTB domain binding to PtdIns at 25°C.

Ligand	*K_d_* (μM)	Δ*H* (kJ/mol)	−TΔ*S* (kJ/mol)	Δ*G* (kJ/mol)
PtdIns_(4)_P_1_	95.00	1.51	−24.46	−22.95
PtdIns_(4,5)_P_2_	125.1	1.94	−24.21	−22.27
PtdIns_(5)_P_1_	No binding			

**Table 2:** Summary of the thermodynamic data for Shc interaction with *β*_3_ integrin derived mono- and bi-tyrosine-phosphorylated peptides acquired from the ITC measurements at two temperature points using full length Shc and the PTB domain constructs.

Shc Construct	Ligand	T (°C)	*K_d_* (μM)	Δ*H* (kJ/mol)	Δ*Cp* (kJ/mol·K)	−TΔ*S* (kJ/mol)	Δ*G* (kJ/mol)
PTB Domain	bi-*β*_3_	25	5.14	−22.98	−0.88	−7.191	−30.17
		12	4.32	−11.52		−17.75	−29.27
	c-*β*_3_	25	0.35	−25.41	−0.09	−11.45	−36.86
		12	1.05	−24.18		−8.446	−32.63
Full Length	bi-*β*_3_	25	2.84	−28.82	−0.96	−2.784	−31.60
		12	5.21	−16.27		−12.55	−28.82
	c-*β*_3_	25	1.33	−7.40	1.04	−26.13	−33.53
		12	0.82	−21.03		−12.17	−33.20

**Table 3:** Summary of the thermodynamic data for Shc interaction with *β*_3_ integrin derived mono- and bi-tyrosine-phosphorylated peptides acquired from the ITC measurements at 12°C in the presence and the absence of Ins_(1,4,5)_P_3_.

Shc Construct	Ligand	*K_d_* (μM)	Δ*H* (kJ/mol)	−TΔ*S* (kJ/mol)	Δ*G* (kJ/mol)
PTB Domain	bi-*β*_3_ bi-*β*_3_ + Ins_(1,4,5)_P_3_	4.32	−11.52	−17.75	−29.27
		3.65	−12.37	−17.30	−29.66
	c-*β*_3_ c-*β*_3_ + Ins_(1,4,5)_P_3_	1.05	−24.18	−8.45	−32.63
		1.12	−23.62	−8.84	−32.46
Full Length	bi-*β*_3_ bi-*β*_3_ + Ins_(1,4,5)_P_3_	5.21	−16.27	−12.55	−28.82
		4.38	−13.87	−15.37	−29.23
	c-*β*_3_ c-*β*_3_ + Ins_(1,4,5)_P_3_	0.82	−21.03	−12.17	−33.20
		0.87	−21.11	−11.96	−33.07

## Abbreviations

PtdIns: Phosphatidylinositol
NMR: Nuclear Magnetic Resonance spectroscopy
ITC: Isothermal Titration Calorimetry
Shc: Src homolog and Collagen homolog
PTB: Phospho-Tyrosine Binding
MAPK: Mitogen-Activated Protein Kinase
*β*_3_CT: *β*_3_ integrin Cytoplasmic Tail
SH2: Src Homology 2
*K_d_*: dissociation constant
bi-*β*_3_: *β*_3_ integrin derived bi-phosphorylated (Y^747^ and Y^759^) peptide
pY: phosphorylated tyrosine
PtdIns_(4)_P_1_: Phosphatidylinositol-4-phosphate
PtdIns_(4,5)_P_2_: Phosphatidylinositol-4,5-diphosphate
Ins_(1,4,5)_P_3_: D-*myo*-Inositol-1,4,5-triphosphate
PtdIns_(5)_P_1_: Phosphatidylinositol-5-phosphate
c-*β*_3_: *β*_3_ integrin derived peptide phosphorylated at C-terminal tyrosine (Y^759^)
SDS-PAGE: Sodium Dodecyl Sulfate Poly-Acrylamide Gel Electrophoresis
^15^N-HSQC: ^1^H-^15^N Heteronuclear Single Quantum Correlation Spectroscopy

## References

[1] Vinogradova O (2002). A Structural Mechanism of Integrin Alpha(IIb)Beta(3) “Inside-Out” Activation as Regulated by Its Cytoplasmic Face. Cell.

[2] Vinogradova O (2004). Membrane-Mediated Structural Transitions at the Cytoplasmic Face during Integrin Activation. Proceedings of the National Academy of Sciences USA.

[3] Deshmukh L (2011). Tyrosine Phosphorylation as a Conformational Switch: A Case Study of Integrin Beta3 Cytoplasmic Tail. Journal of Biological Chemistry.

[4] Deshmukh L, Gorbatyuk V, Vinogradova O (2010). Integrin Beta3 Phosphorylation Dictates Its Complex with Shc PTB Domain. Journal of Biological Chemistry.

[5] Schaffner-Reckinger E (1998). Distinct Involvement of Beta3 Integrin Cytoplasmic Domain Tyrosine Residues 747 and 759 in Integrin-Mediated Cytoskeletal Assembly and Phosphotyrosine Signaling. Journal of Biological Chemistry.

[6] Jenkins AL (1998). Tyrosine Phosphorylation of the Beta3 Cytoplasmic Domain Mediates Integrin-Cytoskeletal Interactions. Journal of Biological Chemistry.

[7] Phillips DR (2001). Integrin Tyrosine Phosphorylation in Platelet Signaling. Current Opinion in Cell Biology.

[8] Law DA (1999). Integrin Cytoplasmic Tyrosine Motif Is Required for Outside-In AlphaIIbbeta3 Signalling and Platelet Function. Nature.

[9] Cowan KJ, Law DA, Phillips DR (2000). Identification of Shc as the Primary Protein Binding to the Tyrosine-Phosphorylated Beta3 Subunit of Alpha IIbbeta 3 during Outside-In Integrin Platelet Signaling. Journal of Biological Chemistry.

[10] Higashi T (2004). Direct Demonstration of Involvement of the Adaptor Protein ShcA in the Regulation of Ca^2+^-Induced Platelet Aggregation. Biochemical and Biophysical Research Communications.

[11] Pelicci G, Lanfrancone L, Grignani F, McGlade J, Cavallo F, Forni G (1992). A Novel Transforming Protein (SHC) with an SH2 Domain Is Implicated in Mitogenic Signal Transduction. Cell.

[12] Ravichandran KS (2001). Signaling via Shc Family Adapter Proteins. Oncogene.

[13] Rameh LE, Chen CS, Cantley LC (1995). Phosphatidylinositol (3, 4, 5)P_3_ Interacts with SH2 Domains and Modulates PI 3-Kinase Association with Tyrosine-Phosphorylated Proteins. Cell.

[14] Balla T (2005). Inositol-Lipid Binding Motifs: Signal Integrators through Protein-Lipid and Protein-Protein Interactions. Journal of Cell Science.

[15] Zhou MM, Ravichandran KS, Olejniczak ET, Petros AM, Meadows RP, Sattler M (1995). Structure and Ligand Recognition of the Phosphotyrosine Binding Domain of Shc. Nature.

[16] DiNitto JP, Lambright DG (2006). Membrane and Juxtamembrane Targeting by PH and PTB Domains. Biochimica et Biophysica Acta.

[17] Farooq A, Zhou MM (2004). PTB or Not to Be: Promiscuous, Tolerant and Bizarro Domains Come of Age. IUBMB Life.

[18] Farooq A, Plotnikova O, Zeng L, Zhou MM (1999). Phosphotyrosine Binding Domains of Shc and Insulin Receptor Substrate 1 Recognize the NP*X*pY Motif in a Thermodynamically Distinct Manner. The Journal of Biological Chemistry.

[19] Zhou MM, Harlan JE, Wade WS, Crosby S, Ravichandran KS, Burakoff SJ, Fesik SW (1995). Binding Affinities of Tyrosine-Phosphorylated Peptides to the COOH-Terminal SH2 and NH_2_-Terminal Phosphotyrosine Binding Domains of Shc. The Journal of Biological Chemistry.

[20] Ravichandran KS, Zhou MM, Pratt JC, Harlan JE, Walk SF, Fesik SW, Burakoff SJ (1997). Evidence for a Requirement for both Phospholipid and Phosphotyrosine Binding via the Shc Phosphotyrosine-Binding Domain *in Vivo*. Molecular and Cellular Biology.

[21] George R, Schuller AC, Harris R, Ladbury JE (2008). A Phosphorylation-Dependent Gating Mechanism Controls the SH2 Domain Interactions of the Shc Adaptor Protein. Journal of Molecular Biology.

[22] Delaglio F, Grzesiek S, Vuister GW, Zhu G, Pfeifer J, Bax A (1995). NMRPipe: A Multidimensional Spectral Processing System Based on UNIX Pipes. Journal of Biomolecular NMR.

[23] Vranken WF, Boucher W, Stevens TJ, Fogh RH, Pajon A, Llinas M (2005). The CCPN Data Model for NMR Spectroscopy: Development of a Software Pipeline. Proteins.

[24] Vinogradova O, Qin J (2012). NMR as a Unique Tool in Assessment and Complex Determination of Weak Protein-Protein Interactions. Topics in Current Chemistry.

[25] Perozzo R, Folkers G, Scapozza L (2004). Thermodynamics of Protein-Ligand Interactions: History, Presence, and Future Aspects. Journal of Receptors and Signal Transduction.

[26] Katyal P, Puthenveetil R, Vinogradova O (2013). Structural Insights into the Recognition of *β*3 Integrin Cytoplasmic Tail by SH3 Domain of Src Kinase. Protein Science.

[27] Ursini-Siegel J, Hardy WR, Zuo D, Lam SH, Sanguin-Gendreau V, Cardiff RD (2008). ShcA Signalling Is Essential for Tumour Progression in Mouse Models of Human Breast Cancer. EMBO Journal.

[28] Pettersen EF, Goddard TD, Huang CC, Couch GS, Greenblatt DM, Meng EC, Ferrin TE (2004). UCSF Chimera—A Visualization System for Exploratory Research and Analysis. Journal of Computational Chemistry.

[29] Gurtovenko AA, Vattulainen I (2007). Lipid Transmembrane Asymmetry and Intrinsic Membrane Potential: Two Sides of the Same Coin. Journal of the American Chemical Society.

