# Supplementary material for: Phospho-Tyrosine(s) vs. Phosphatidylinositol Binding in Shc Mediated Integrin Signaling
Source: Am J Mol Biol. Author manuscript; Available in PMC 2015 Apr 15. (PMC4397963; doi:10.4236/ajmb.2015.52003)
Supplement: Supplementary file 1 [file NIHMS675211-supplement-supplement_1.pdf]

Supplementary Materials

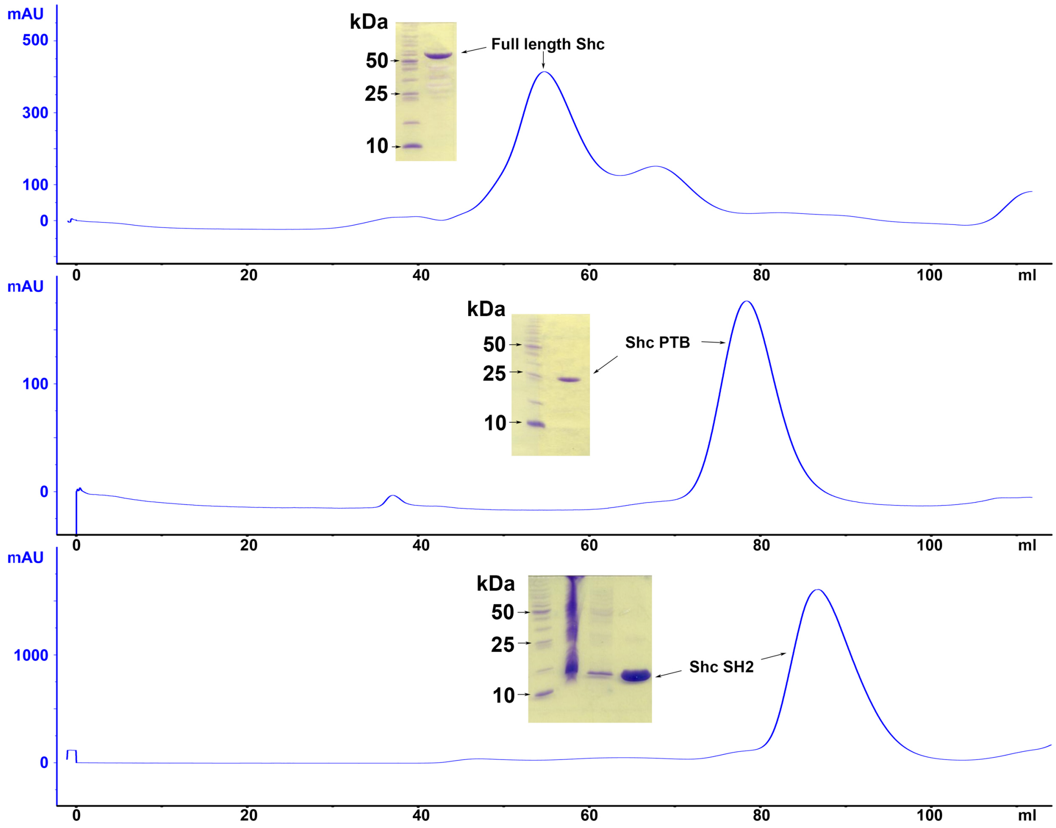

Figure S1. Chromatograms from size-exclusion chromatography obtained on a Superdex-75 column and SDS-PAGE gel pictures of full-length Shc, Shc PTB domain and SH2 domain. All three recombinant proteins are shown in monomeric state with a good purity after the purification process.

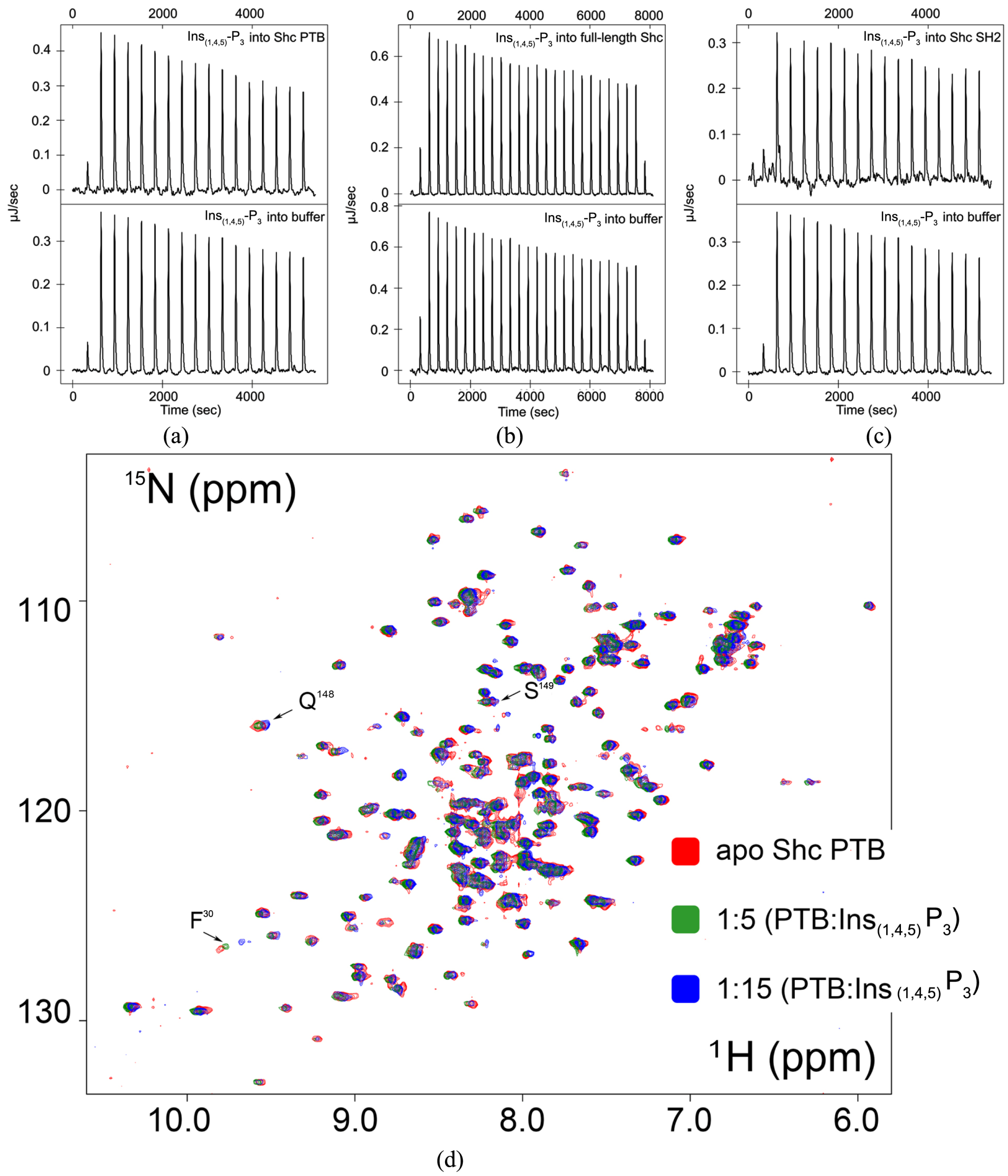

Figure S2. (a) $Ins_{(1,4,5)}P_3$ does not show to bind to Shc PTB domain at 25˚C in ITC; (b) $Ins_{(1,4,5)}P_3$ does not show to bind to full length Shc at 25˚C in ITC; (c) $Ins_{(1,4,5)}P_3$ does not show to bind to SH2 domain at 25˚C in ITC; (d) ^{15}N-HSQC titration of PTB domain by $Ins_{(1,4,5)}P_3$. The effect from $Ins_{(1,4,5)}P_3$ only show up at a high PTB-to-$Ins_{(1,4,5)}P_3$ ratio (1:15) and is less pronounced compared to $PtdIns_{(4,5)}P_2$.

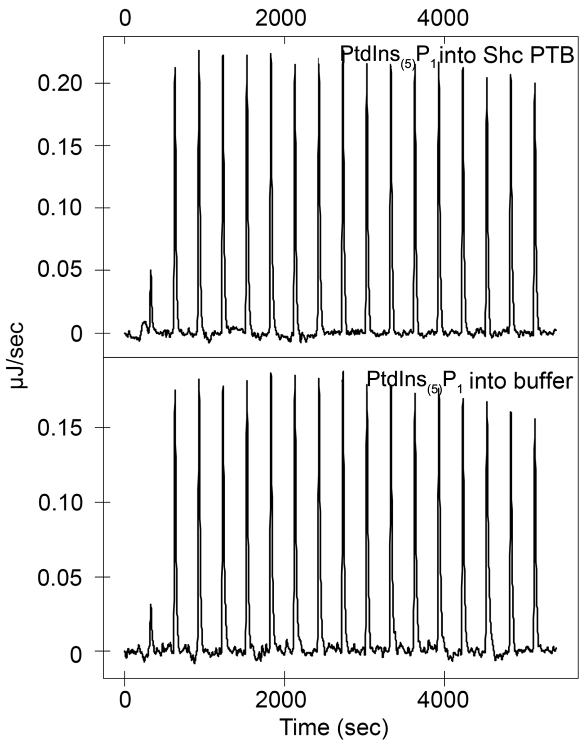

Figure S3. $PtdIns_{(5)}P_1$ does not show to bind to Shc PTB domain at 25˚C in ITC.

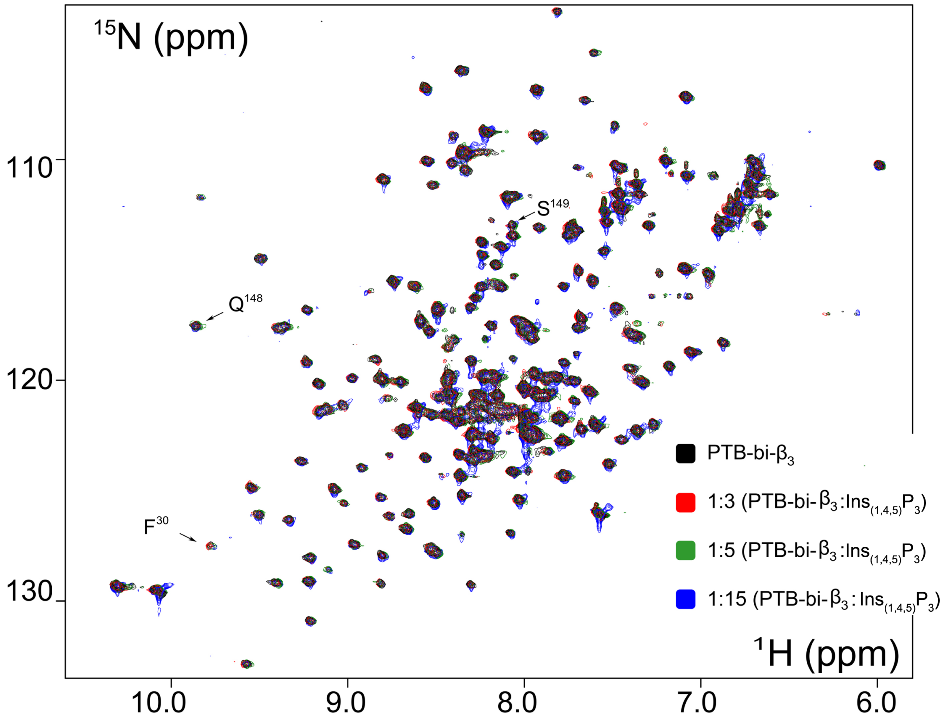

Figure S4. ^{15}N-HSQC spectra of Shc PTB pre-satured by $Ins_{(1,4,5)}P_3$ (at protein-PtdIns ratios of 1:0, 1:3, 1:5 and 1:15) with bi-β_3 added (at a 1:2 protein-to-peptide ratio). The lack of difference in the overlaid spectra indicates that integrin tyrosine(s)-phosphorylated β_3 integrin CTs can easily replace PtdIns from the overlapping binding sites on the Shc surface.
